# Alarming trends in Tobacco use in high school Tunisian adolescents: MedSPAD2013-2021: Ahlem Silini

**DOI:** 10.1093/eurpub/ckac131.216

**Published:** 2022-10-25

**Authors:** A Silini, S Rejaibi, M Zid, N Zoghlami, R Mallekh, I Ben Slema, S Ben Youssef, M Zribi, N Ben Salah, H Aounallah-Skhiri

**Affiliations:** 1 Epidemiology Department, The National Health Institute, Tunis, Tunisia; 2 Medical Faculty of Medicine, Tunis El Manar University, Tunis, Tunisia; 3 Nutrition Surveillance and Epidemiology, SURVEN, Research Laboratory, Tunis, Tunisia; 4 Intensive Care Unit Department, Center for Urgent Medical Assistance, Tunis, Tunisia

## Abstract

**Background:**

Tobacco use is a global health concern, with smoking initiation often beginning in adolescence. In Tunisia, published data assessing trends of tobacco consumption at the national level only concerns middle school students aged 13 to 15 years. No data related to high school students are however published yet. We aimed to determine tobacco smoking prevalence in Tunisian adolescents and assess trends from 2013 to 2021.

**Methods:**

Pooled data from three Mediterranean school surveyson alcohol and other drugs (MedSPAD surveys: 2013, 2017, and 2021) were used. Based on three-stage stratification sampling method, teenagers aged between 15 and 17 years, were enrolled. Were not included students enrolled in vocational training centers and out-of-school adolescents. Data collection was performed using a self-administered standardized questionnaire assessing socio-demographic characteristics and risky behaviours. We studied weighted prevalence estimates of cigarettes and water pipe (WP) smoking. All statistical analysis, including trend analysis, were performed with STATA software.

**Results:**

A total of 14.723 students were enrolled with sex ratio (M/F) equal to 0.6 and mean age of 16.2±0.8 years. The prevalence of cigarette smoking increased from 17.8%, to 20.2% then to 24.7% for 2013, 2017 and 2021, respectively. As for WP smoking, its prevalence increased from 14.8%, to 16.7% then to 19.9% for 2013, 2017 and 2021, respectively. Trends analysis concluded to significant increase over study period for cigarettes and WP smoking (p < 10-3). The increase was most alarming for girls regarding WP smoking (consistent increase over the study period).

**Conclusions:**

Our findings underscore the alarming increasing trend for different forms of tobacco smoking among Tunisian youth. It is therefore crucial to strengthen tobacco control measures among young adolescents in order to counteract the tobacco industry’s expanding marketing of new products primarily targeting this population.

**Key messages:**

• The prevalence of cigarette and water pipe smoking increased significantly among high school Tunisian adolescents from 2013 to 2021.

• A better commitment to the implementation of MPOWER measures for tobacco control is therefore urgent.

